# Detecting Shipping Container Impacts with Vertical Cell Guides inside Container Ships during Handling Operations

**DOI:** 10.3390/s22072752

**Published:** 2022-04-02

**Authors:** Sergej Jakovlev, Tomas Eglynas, Miroslav Voznak, Mindaugas Jusis, Pavol Partila, Jaromir Tovarek, Valdas Jankunas

**Affiliations:** 1 Marine Research Institute, Klaipeda University, Herkaus Manto Str. 84, LT-92294 Klaipeda, Lithuania; tomas.eglynas@ku.lt (T.E.); miroslav.voznak@ku.lt (M.V.); mindaugas.jusis@ku.lt (M.J.); pavol.partila@ku.lt (P.P.); jaromir.tovarek@ku.lt (J.T.); valdas.jankunas@ku.lt (V.J.); 2 Department of Telecommunications, VSB—Technical University of Ostrava, 17. Listopadu 2172/15, 70800 Ostrava, Czech Republic

**Keywords:** signal processing, data fusion, container handling, IoT sensor

## Abstract

Due to the mechanical nature of container handling operations, as well as natural factors, container and handling infrastructure suffers various types of damage during use, especially within the tight and enclosed environments of a ship’s hull. In this operational environment, it is critical to detect any sort of physical impacts between the vertical cell guides of the ship’s hull and the container. Currently, an inspection of impacts and evaluation of any consequences is performed manually, via visual inspection processes. This process is time-consuming and relies on the technical expertise of the personnel involved. In this paper, we propose a five-step impact-detection methodology (IDM), intended to detect only the most significant impact events based on acceleration data. We conducted real measurements in a container terminal using a sensory device placed on the spreader of the quay crane. The proposed solution identified an average of 12.8 container impacts with the vertical cell guides during common handling operations. In addition, the results indicate that the presented IDM can be used to recognize repeated impacts in the same space of each bay of the ship, and can be used as a decision support tool for predictive maintenance systems.

## 1. Introduction

Port operators and shipping companies constantly face several serious problems related to shipping container handling in the port environment while transporting shipping containers and performing handling operations with port infrastructure [1]. From an engineering perspective, the most common handling mistakes occur during the transportation of shipping containers from container ships [2]. On container ships, the position of containers is identified by a specific bay-row-tier coordinate system. This system is identical on almost all ships. The bays illustrate the cross-sections of the ship and are numbered from bow to stern. The rows run the length of the ship and are numbered from the middle of the ship outwards. Meanwhile, the tiers represent the layers of containers. Containers are raised and lowered within the holds with the aid of vertical cell guides. The placement of the shipping containers is performed by quay cranes (QC) and this process is known as handling the container. Depending on the structure, container ships can have several holds, specific spaces on the ship that can hold containers vertically, and which are divided into two parts depending on whether they are on-deck or under-deck (i.e., in the hold) within the ship’s hull. Containers have exact cells, i.e., positions in these bays, along the ship’s length [3]. Vertical cell guides are relatively simple structures that are serve to guide the containers inside the holds during their lowering and extraction. Without them, it would be impossible to place containers steadily and accurately in due time. However, they can be damaged quite easily by a few localized impacts from heavy containers if these are swinging without strict control inside the under-deck sections of the ship, halting the entire handling process for the whole ship. Even in such small spaces, with only 5 cm of free space from each side, a 30-ton container has enough dynamics to cause serious problems and damage both the metal infrastructure of the ship and the container itself. If the impacts occur repeatedly in the same places during a long period of operational time, this can cause structural damage to the ship, as well as to valuable goods. Without knowing where these impacts occur, it is difficult to mitigate against these risks of structural damage and cargo loss. Therefore, precise smart monitoring of these security events is a critical issue, which must be solved by employing various technological and methodological solutions.

**Figure 1:**
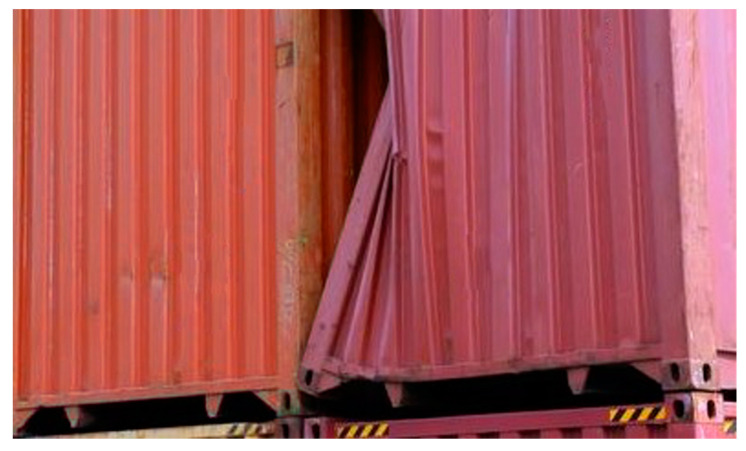
Example of a damaged marine container.

Security events related to container impacts with the vertical cell guides mostly occur naturally, due to many external factors, yet a small percentage of these events occur due to illegal or unprofessional actions. Containers tend to bend and shake heavily when subjected to such impacts, thus increasing the chances of breaching their integrity and allowing moisture, gases, or biological agents to enter and cause severe damage to cargo. The possibility of a critical impact is mostly random and is very difficult to detect in a real-time manner, even utilizing the smartest and the fastest methods available. Near real-time solutions are constantly introduced to the market and many intrusion scenarios are being tested by engineers worldwide. However, in real-life applications, trained operators still use ISO-regulated methods of visual detection, relying on their experience and supporting their decisions with the help of other personnel onboard the ship. In normal situations, structural damage can occur on a side wall, on the door, on the corners, or over multiple sections (see Figure 1) at any given moment, due to natural factors, unseen and undetected obstructions, poorly performed handling procedures, or simply due to corrosion of the container walls.

It should be noted that in such a harsh environment, where every object has its own dynamic properties, such as the constant movement of the ship near the berth, unpredictable wind gusts, operator actions, etc., it is extremely difficult to control the handling process with optimized coordination and to limit unnecessary contacts with other metal structures surrounding the handled container. Bad stowage can cause damage to multiple containers onboard the ship. Damage also occurs when heavyweight containers find their way into the upper tiers of container stacks on decks. The damage can be minimal or large enough to stop the entire transportation process within a single hold, sometimes destroying goods completely.

**Figure 2:**
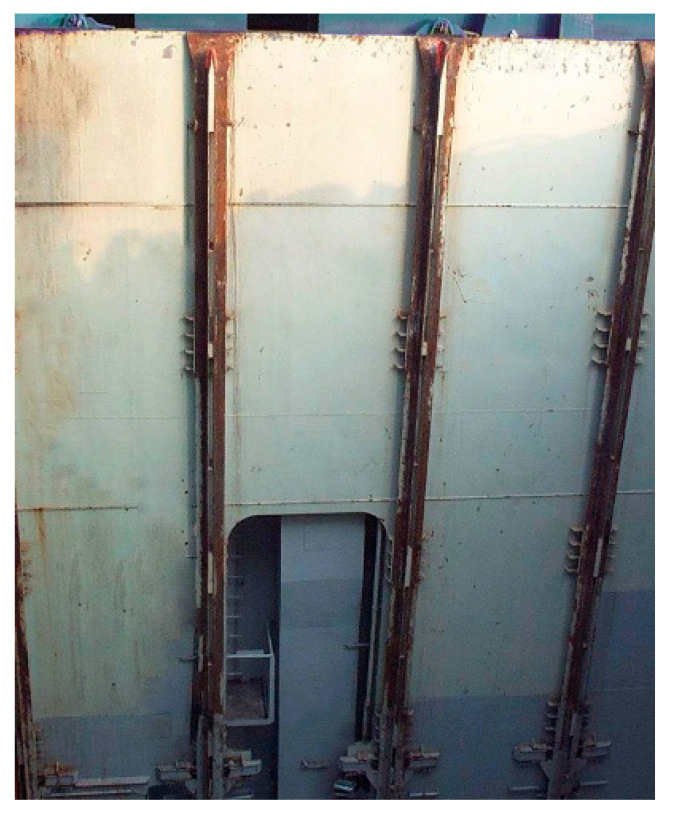
Example of the vertical cell guides in the inner hull of container ship representing the hold.

Containers are secured with twist-locks, which lock them down to the deck of the ship. Lashing rods and turnbuckles are used to provide additional strength to help secure the containers in place. Custom corner fittings, wheels, and mountings are also used occasionally. Vertical placement of containers within the inner parts of the individual holds of the ship is performed using vertical cell guides (see Figure 2). One of the most persistent problems experienced onboard container ships is container sway during their handling by the QC inside the ship’s inner hold, as the containers are moving through the vertical cell guides. This can result in serious downtime whilst damaged sections of the guide are replaced, and later legal actions.

**Figure 3:**
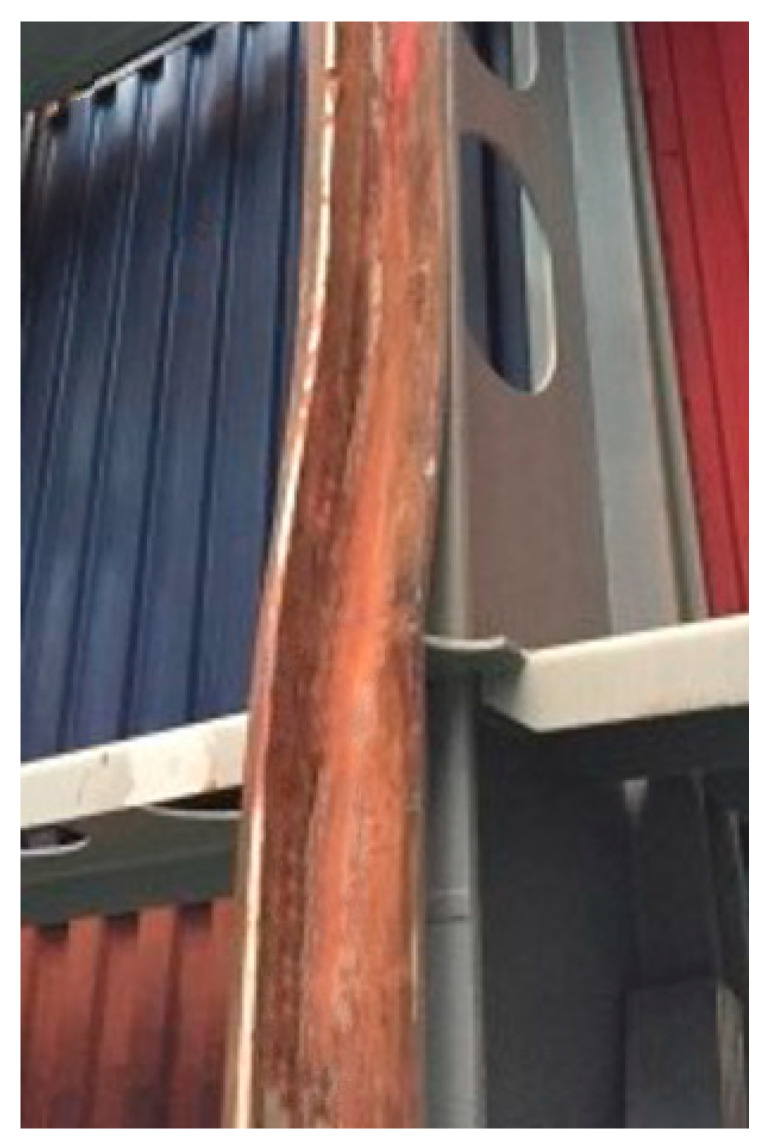
Example of a damaged vertical cell guide.

At this point, the biggest challenge is the security and efficiency of the container placement procedure. If performed inappropriately and without strict safety regulations, this could damage not only the container but also the guides, endangering the personnel working nearby (see Figure 3).

Container movement along the vertical cell guides is performed using long cables, attached to the spreader of the QC. For this reason, the process itself is nonlinear; the observed motion of the spreader and the attached container follows a nonlinear state equation, more similar to a complex sinusoidal dependency, due to the natural swinging of the two attached bodies in free space. From a strict engineering point of view, control and prediction of such heavy machinery movement in real-time is a very difficult and mostly unoptimized task for port scheduling systems. However, simpler and more practical solutions to this problem may prove far more effective than mainstream methods.

Damage to the vertical cell guides can take diverse forms, and the actual damage may be of a single type or a multitype combination, depending on whether the deformation of the structure is caused by the sliding of the container or a severe impact. The variety and the severity of the damage can vary for each case. Detecting of specific damage types is challenging when performed using either visual inspection or by means of digitized inspection. The main cause of damage to the vertical cell guides may be a result of physical wear, wrongly performed container handling operations, or other human error factors. However, most of these events are difficult to predict due to the large volumes of containers being handled each day. It is important to accurately identify any damaged areas in due time and to identify the true cause of the damage, whether this occurs during the carriage of the containers by sea or container handling in maritime ports.

Climatic conditions impact the structural integrity of the vertical cell guides, introducing wear due to corrosion. On the other hand, mechanical damage can be classified into two main categories:

- Static—as the result of the impacts of containers on the guides due to the force of gravity;
- Dynamic—as the result of accelerations from physical phenomena in the process of container handling within the holds of the ship.

In this paper, we propose a possible impact-detection methodology based on signal filtering techniques whilst eliminating natural causes from real impact occurrences. The rest of this article is structured as follows: Section 2 introduces the recent advancements in the areas of transportation, control, and management of containers in ports; Section 3 elaborates on the construction details of the sensor device framework and the measurement environment; Section 4 describes the step-by-step event detection methodology; Section 5 discusses the results of this detection method study and the future direction of this research; and finally, Section 6 presents the conclusions.

## 2. Review of Recent Advancements

Ports utilize large-scale inspection devices, heavy-machinery and skilled personnel to detect any damage aboard ships and to the containers [4]. The damage detection is performed manually by visual inspection on-site, after a critical event or during routine checks [5]. If the inspection of the ship is performed regularly, the risk of vertical cell guide damage caused by containers in the later transportation process can be avoided. However, this detection method is time-consuming and labor-intensive, and places high demands on specialized personnel working aboard the ships [6]. Many researchers are studying methods and developing complex sensory systems [7] to assist or replace manual infrastructure inspection aboard ships, including optical devices, laser scanners, etc. However, the problem of vertical cell guide wear is not well considered in the literature [8]. In addition, the research literature does not consider the problem of the jamming of containers inside the holds during the handling process, which can cause serious damage to the ship’s hull, the containers, and cargo handling equipment, halting the entire handling operation.

Signal-processing algorithms applicable to real-world engineering solutions are a hotspot of data science research [9,10]. Although the related research is in the development stage, due to the aforementioned reasons, there is much scientific literature on this common research direction in the sub-sections of transportation [11,12] and computer science [13,14,15].

The current research on this topic shows the limitations of automated methods for detecting damage to container and handling infrastructure in the context of container shipping, and suggests trends for future research. Firstly, fully automated and smartly digitized damage detection methods have not been applied on a large scale in the global supply chain, are limited by the operational environment, and lack standardized solutions [12]. Secondly, the proposed impact-detection methods highlight those features of a more complex multi-feature methodology for ship and container damage detection that are relative to the general and aforementioned container handling operations. The inclusion of a broader spectrum of parameters and sensory units could potentially increase the viability of these processes and enable more accurate estimations [16]. In addition, most of the existing research focuses on damage detection based on visual inspection, using real image datasets produced using visual inspection tools, collected by the repair personnel, related port authorities, and logistics companies [17]. However, most of the scientific community whose work focuses on damage detection systems for logistics does not have free access to such datasets, as most experiments are forbidden in the port environment for security and legal reasons. The lack of unified container and handling infrastructure datasets makes it extremely difficult for scientists and engineers to conduct objective experiments and comparison tests of various existing algorithms and sensory systems under uniform conditions.

Regarding the safety and security of the containers and the cargo, a few research efforts have been reported in the literature [18]. However, none of the previous works discuss the application of acceleration sensors to detect container impacts in the inner parts of the ship during handling operations. The patterns of the container sway and natural vibrations of the metal structures are sensitive to many factors, such as the materials, the type and shape of the infrastructure, the speed of the handling procedures, the mass of the container, and the physical characteristics of the suspension system, along with the inclusions of the human factor and the general dimensionality of the test site. Moreover, the acceleration signal patterns can be valuable for forensics, i.e., the patterns can reveal if the container has been handled with care during the procedures, or if there have been collisions with the vertical cell guides aboard the ship, causing unnatural sway [19,20,21], sliding effects, and other modes of stability loss during transportation aboard the ship and beyond. However, the effective detection of genuine events is challenging, as the measured signals may be disrupted due to the handling processes or wireless data transmission losses [22], and containers may manifest multiple concurrent modes of stability loss during their placement within the inner holds.

The recent literature contains many technological solutions for minimizing the number of real damage event occurrences. Smart Digital Technologies (SDT) integrated into the logistic processes of the port are becoming increasingly decisive due to the need for policymakers, urban planners, governmental administrators, managers, transport operators, related port authorities, and shipping companies to develop intelligently digitalized and overall sustainable logistic processes for the entire port and beyond [23,24,25]. To cope with the intense datafication and globalization of trade on a global scale [26], mostly using shipping containers, new data-driven approaches are being developed [27,28] to promote intelligent and sustainable development of digital and secure logistics [29,30]. The aim of this research further strengthens the research potential of the engineering community and participating organizations in the area of Smart Logistics [31], utilizing the new generation of Information technologies (IT), such as Cloud computing, the Industrial Internet of Things (IIoT) [32], 5G/LTE, and Big Data analytics [33]. The intention is to deepen our strategic knowledge of security and safety problems associated with Logistics 4.0, which entails highly flexible and adaptable logistics supply networks along with a variety of structural ICT solutions and multi-functional high-level security processes.

Container handling operations within ports can be generally classified into the following two categories:

- Planning methodologies, used for strategic/tactical purposes;
- Control techniques, used for operational purposes.

As for the problem of planning, the main aim is to develop optimized online systems to govern the motion and transportation of containers in terms of routing, sequencing, and scheduling problems [34]. From the planning perspective, new data driven-solutions can have a critical influence on the efficiency of operations, including the security of containers, personnel, and handling equipment, but the data must provide real-time information for the predictive maintenance of the ship’s infrastructure, including a signaling tool to inform repair units to check the containers and vertical cell guides of the holds in the event of a significant impact, or a collective data set that provides statistical evidence of multiple impacts to the same areas. If the management procedures include sophisticated algorithms intended to optimize resources, operation times, etc., then any form of disturbance [35], including damages or security-related events, can seriously impact planning, and can even cause chaos among the personnel.

Regarding the control operations, Predictive Model Controllers (MPC) are widely used to control heavy machinery and they tend to perform quite well [36]. The application of AI methods in these MPC control systems [1,37,38] tends to result in more efficient performance compared to regular methods and on-site human operators. However, these kinds of systems are mainly developed to be used as supporting tools in the form of decision support programs that highlight the optimum speed of transportation and the best movement patterns for containers and other means of transport within the terminal. These systems rely on data and process synchronization systems that connect all means of transport within the container terminal, ranging from diesel trucks to autonomous electrical trucks and various port cranes (QC, and YC) [39,40]. Management of these processes is performed using smart software solutions with smart Edge-Cloud infrastructures [41]. IIoT sensors with efficient power management solutions work to receive data from shipping containers, detecting their position, movement trends, weather parameters, inner cargo dynamics, etc., [42], with the aim of minimizing delays caused by any disruption and disturbances. However, ships tend to have complex dynamics due to mechanical, natural, and other disturbances. Threats to the containers arise during all means of transportation, but major impacts occur during QC handling of containers aboard container ships.

There is no doubt that controlling these multiple processes is an optimization task too great to be solved by any individual management or control system. If no opportunity exists to install complex smart controllers within the cranes, then a simplified decision support system can be implemented, supporting the control procedures, and suggesting control patterns by detecting impacts, registering relevant events, and calculating possible risks. However, optimized maneuvering of the container inside the holds of the ship is a serious and demanding task even for operators with years of experience, due to the extremely narrow spaces for maneuvering. That is why detection of all “true” impacts is a must for any practical decision support system adapted for managerial decision support and risk assessments [43,44].

It is also worth mentioning the problems posed by the integration of shipping containers with different transportation modes, the optimization of resources, and the use of specialized modeling tools to ensure the operational performance of intermodal transportation [45]. When containers are positioned and lowered through the vertical cell guides, they tend to impact the vertical cell guides quite often and with varying force. To stabilize the process, many control solutions have been developed over the years, yet they all have downfalls related to the complexity of the dynamics of the ship, the quay crane, the trucks, and all other means of transportation [46,47,48]. Optimization of the handling processes is a priority for all logistics companies and it is certainly a hot topic in the current literature [49,50,51,52], yet optimization of container handling processes only rarely takes into account the damage perspective [8], which relates to the physical impacts of containers and the surrounding infrastructure. On the other hand, novel data analytics methods are being developed along with the rising popularity of embedded IoT devices [53], which are capable of delivering adequate solutions for decision-critical operations [54]. Their applicability and effectiveness in solving specific monitoring and local decision support tasks are astonishing [55] compared to conventional methods. Most of these systems rely on simple sensory signal filtering methods [10], while others use AI-based solutions [21,56], deep learning [57], and other machine learning methods [58] to filter noises in the raw data [59], classifying events [60], detecting patterns [61], eliminating cyber threats in communication networks [60,62,63], compressing the data [64], etc.

## 3. Description of the Measurement Environment and the Sensor Device

To support the proposed methodology, an experimental case study was performed using a custom real-time data acquisition IoT sensor developed and tested in a container terminal in Klaipeda port at the LKAB “Smeltė“ container terminal. The data samples were collected every 100 ms via the sensory unit, allowing the system to collect real-time information on movements and impacts. Beacon intervals and data rates were 100 milliseconds and 250 kbit/s, respectively. The previous measurement results were sent to the server and the user, along with a blank alert message in the packet, forming a historical log remotely and in the SD card of the device as a reserve.

Each new packet was formed from a *t*−1 measurement, allowing the system to form the packet and send it to the user, thus having a 100 ms delay. Edge computing capability was established to perform inner analysis of the acquired data samples, described in the next section. The end-node device system consisted of:

- A data transmission unit;
- A SINDT-232 Digital accelerometer with high-stability 200 Hz MPU6050 3-Axis acceleration, and a 0.05-degree accuracy. Additional parameters were as follows: Current: <40 mA; Voltage: 5–36 V; Data output frequency: 0.2–200 Hz; Baud Rate: 4800-961200 (adjustable); Working Temperature: −40 ℃~+85 ℃; Range: Acceleration (±16 g);
- An edge computing unit based on Raspberry Pi 4 (four ARM A72 1.5 GHz cores, 8 Gb of RAM) used to analyze the signals, form alert messages, and write the connected sensors data at high speeds to the provided 128 Gb SD UHS-I card;
- An inner 6000 mAh battery;
- A common enclosure for the electronics built with IP65 protection.

**Figure 4:**
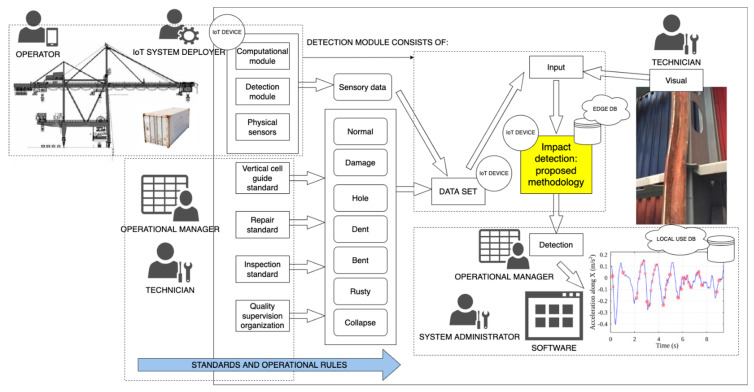
The high-level schematic of the process of data collection using the IoT sensor for vertical cell guide damage detection, using the frequency threshold detection algorithm and the visual inspection policy.

Figure 4 demonstrates the framework of the IoT system, the operational environment, the actors, and the data sources used within this experimental setup. The research method framework for vertical cell guide damage (impact) detection combined acceleration signal processing using a frequency threshold detection algorithm, a visual inspection policy, operational standards, and electronics.

Here, each IoT sensor is a combination of inner electronics, wireless communication and data storage protocols, and a specified computational mechanism, the processor, where the sensor data manipulations are performed using introduced software logic–the developed detection methodology—with step-by-step instructions. The person responsible for the IoT is the system deployer, while the administrator manages the data flows within the dedicated software and the locally operated database. Other key members include the operators of the heavy machinery on site, namely the crane operators; the technicians, who are sent to check the situation and to perform the repair works, while also receiving intel about the situation and suggestions on fixing the problems; and, finally, the operational managers, who perform planning tasks for the terminal, managing logistics and handling processes. With the help of the Edge computing paradigm, every single computational unit performs the analysis, and the data are stored locally, eliminating the need for complex wireless or wired communication channels for data acquisition from all sensors. Each IoT sensor computes the acceleration values according to the inner logical rules, saving the data for critical incidents, and sending the filtered results to the operational manager, who in return suggests predictive maintenance of some of the parts of the guides or the containers. This kind of framework minimizes interference from the human factor and increases the chances of getting the real situation data in time. With each new container handling operation, the holds of all the ships are monitored, along with all the containers, improving the rate of impact detection, and increasing the chances of detecting security events.

**Figure 5:**
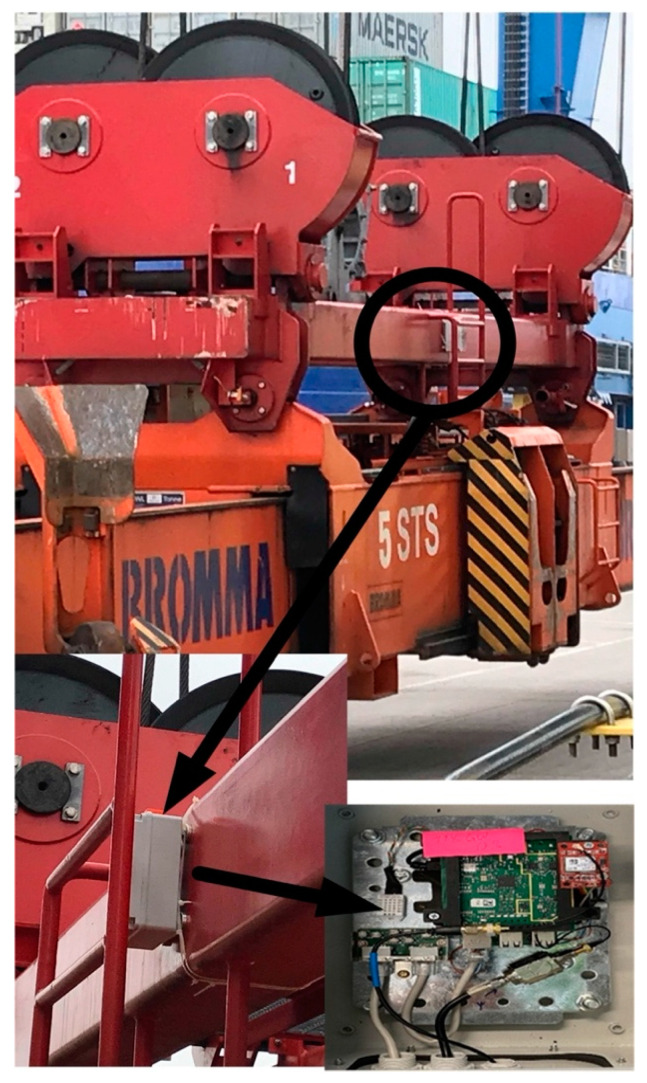
Positioning of the embedded sensory device on the spreader of the QC.

The following Figure 5 demonstrates the exact placement of the device on site and the inner electronics by which it retrieves the data. The sensory device was placed on the spreader of the selected QC in a protected place with magnets and straps holding it in place.

**Figure 6:**
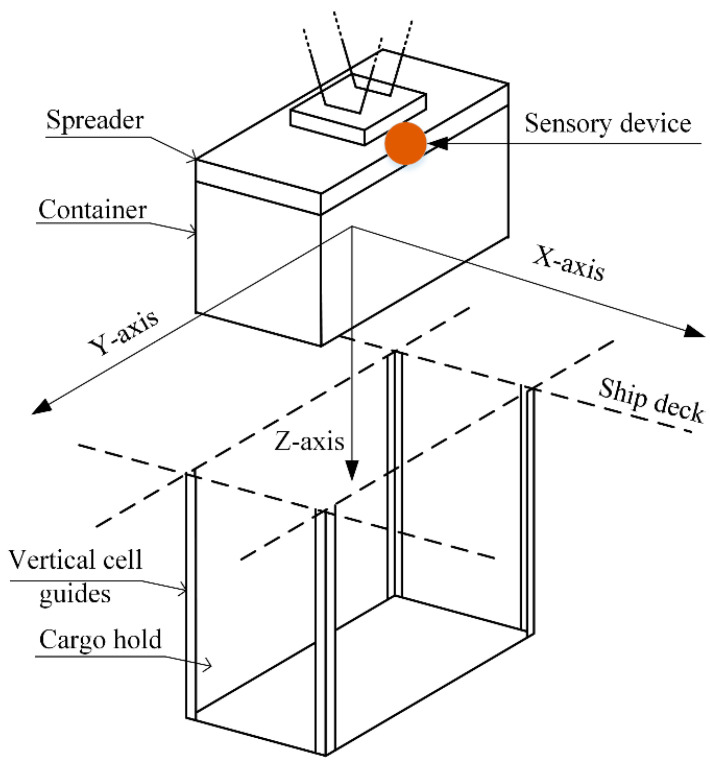
Schematics of container sway detection along with the vertical cell guides.

Data were collected by the sensors starting from the initial vertical movements of the spreader, up till the last moment. Although this would be inefficient within real-world applications, in this research a combination of several key data transfer technologies must be used (LoRa, LTE, 5G, etc.), working in synergy and compensating for the lack of network stability. Schematically, the process of detection can be described as in Figure 6, which indicates the directions of the container movement along all the measured axes.

Containers are placed in stacks inside the ship’s hull, one above the other. The Y-axis marks the movement of the container along the ship’s hull. Throughout the entire handling procedure, the main movement of the container occurs along the X-axis. The mounting point of the sensory device is marked by the red dot.

**Figure 7:**
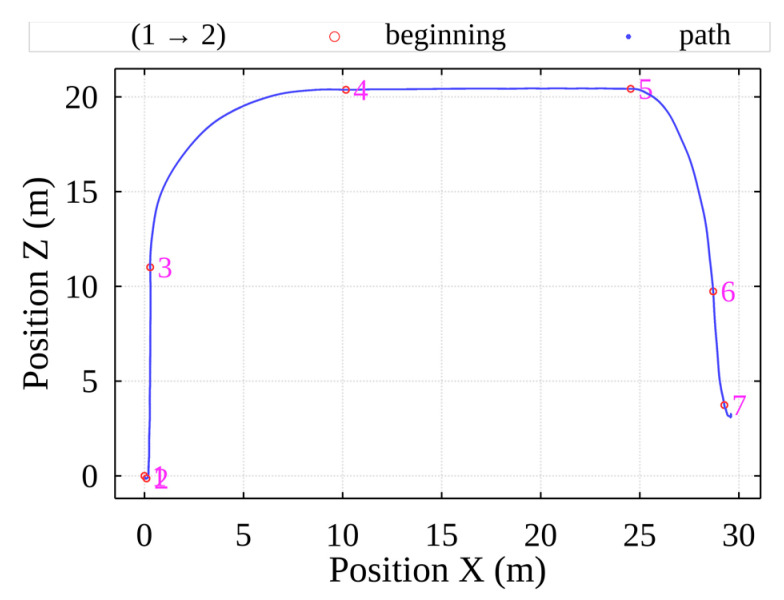
Container transportation path from ship to truck.

The entire process of transporting a single container from the lowest part of the container ship is presented in Figure 7.

Each position from 1 to 7 marks the beginning of a new movement trend, either parallel movements along the ground or vertical movements on both sides of the crane. Thus, the operator uses pre-defined movement and control patterns for each case according to their expertise. Movement patterns between 1 and 2 indicate the process of container hooking by the spreader. Movement patterns between 3 and 4, and between 5 and 6 indicate the sudden change of dynamics with an increased degree of container sway, resulting in unnecessary security issues for the cargo inside and the surrounding infrastructure. Movement patterns between 2 and 3 indicate the movement of the container inside the container ship’s hull. Herein, only the movement between 2 and 3 will be discussed, because the majority of security threats occur there.

**Figure 8:**
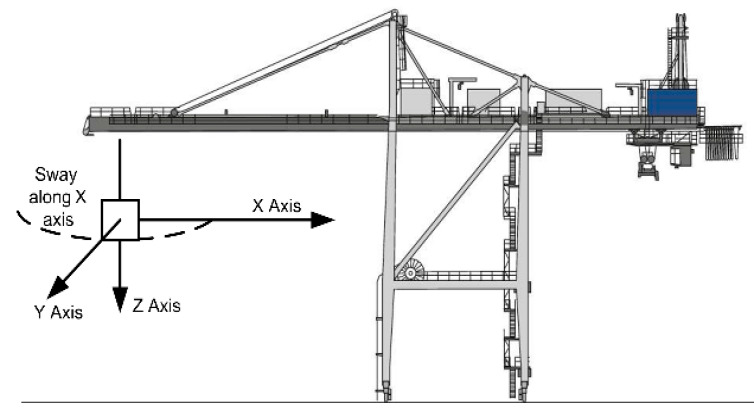
The coordinate system is used to describe the movement of the container.

Figure 8 demonstrates the movement directions of the spreader of the quay crane.

**Figure 9:**
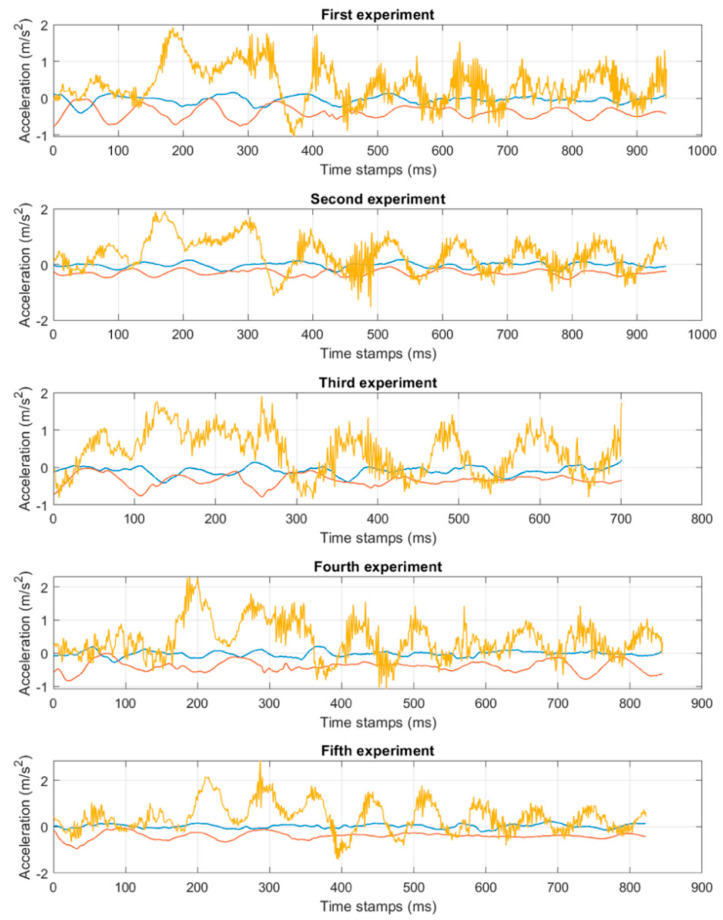
Results of the experimental measurements.

This figure shows the establishment of the coordinate system used in the next section. The movement of the container along the X-axis is critical. Figure 9 demonstrates the results of five measurements, performed by the experimental equipment at the container terminal with the help of the same operator and similar work conditions, and with similar weights of cargo. Each of the five examples demonstrates similar movement patterns along all three movement axes. Therefore, it is concluded that most transportation processes and their dynamic properties have similarities and can be analyzed using a common method. In Figure 9, the blue line indicates acceleration along the X-axis; the red line indicates acceleration along the Y-axis; and the yellow line indicates acceleration along the Z-axis.

**Figure 10:**
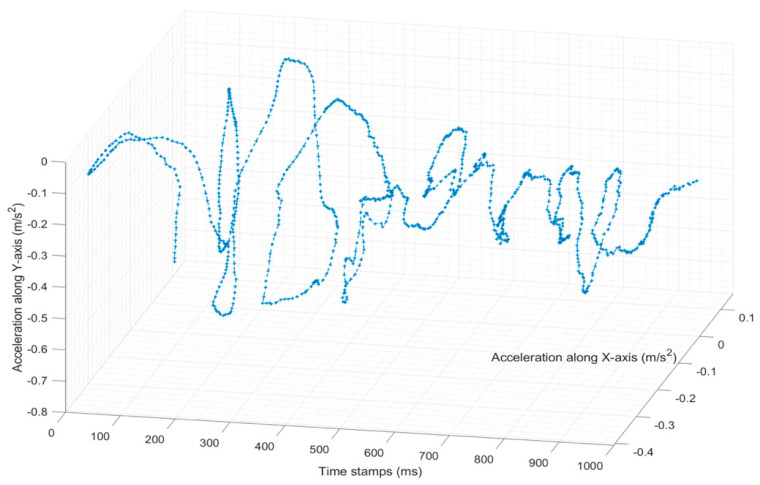
Vertical motion path inside the ship’s hull along the vertical cell guides.

Figure 10 shows the path of the container inside the ship from the first example provided in Figure 9. It can be observed that at the start of the procedure the accelerations values along the X and Y axes rose with each new movement, up until a certain point at around the 400 ms timestamp. They then decreased by a certain degree and reached a stable level, mostly due to constant interactions with the cell guides, sliding and hitting them in many areas.

## 4. Impact Detection Methodology

The proposed methodology is based on the measurement results of the experiment that was performed under real working conditions in the port area. It involved a typical ship loading/unloading procedure. During these experiments, the data logger was attached to the spreader of the QC as described previously, which registered the acceleration values, indicating the possibility of adapting the system within smart containers. The full handling process was recorded during these experiments, but our methodology included only the part of the data during which the transshipment process took place within the hull of the ship.

When a crane transports a container, it oscillates at its natural frequency. Since the amplitude of the oscillation is small compared to the length of the rope holding the container, the oscillation curve in the plane is close to the sinusoid. However, during the loading process, the container is affected by external forces (wind, ship, and wave, operator skills and actions, equipment vibrations, etc.), that complement the container’s spectrum of motion with components of another frequency.

**Figure 11:**
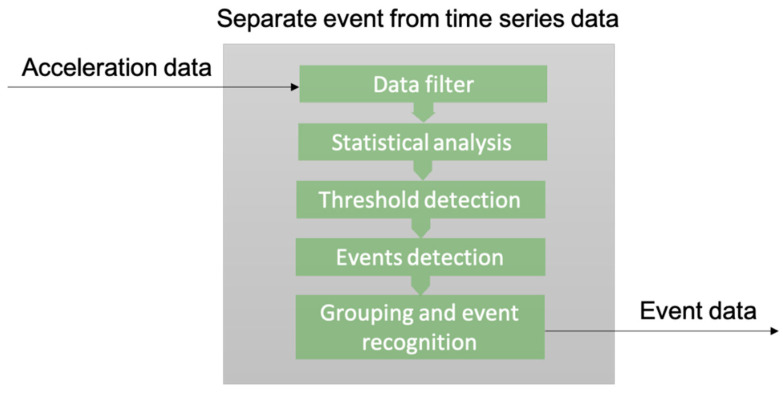
Flowchart of the proposed solution for event data detection algorithm.

The signal processing algorithm is presented in Figure 11, in general terms. This method was developed using historical data for the detection of the true thresholds. The best efficiency was achieved immediately after the loading operation. In this way, an event can be prevented from causing greater damage to the container and the ship’s infrastructure. Although this method must be improved to enable the detection of a single load cycle event, the proposed method may be used for the risk analysis of a vessel in one row on the X-axis. The main advantage of this type of application is the trackability of any actual security events that occur within a given row, before moving to another. This allows for the acquisition of enough data to enable adequate decision-making using the proposed methodology. Each case study must be analyzed separately in future studies.

**Figure 12:**
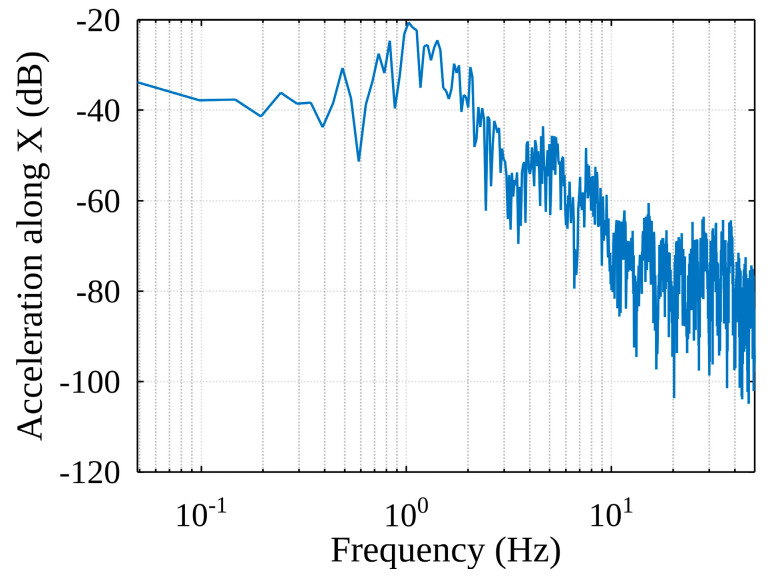
The spectrum of Acceleration along the X-axis (full handling process).

For the whole handling cycle in the spectrum of accelerations along the X-axis of the container (see Figure 12), the peak at 0.6 Hz is likely to be the natural frequency of the pendulum created by the container line. We also see higher frequency components in the spectrum, which are probably the result of external influences.

**Figure 13:**
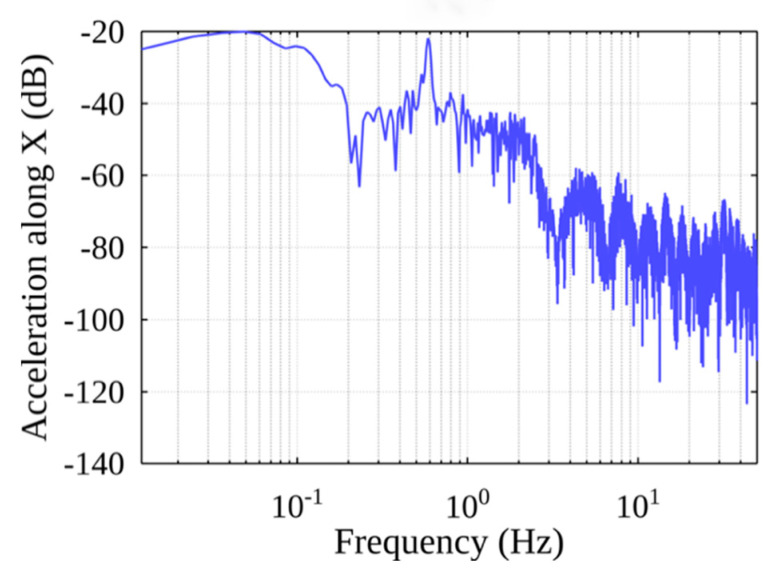
The generalized spectrum of acceleration along the X-axis of the container.

However, to support the methodology in the initial stage of the handling inside the hull of the ship, only data from the vertical movement between Section 2 and Section 3 (Figure 7) were considered. In the summarized acceleration spectrum formed by analyzing the targeted 17 cycles, a similar situation is observed (see Figure 13), namely the range of the dominant component from 0.4 Hz to 2.5 Hz, which is (very likely) generated by the free oscillations of the container.

**Figure 14:**
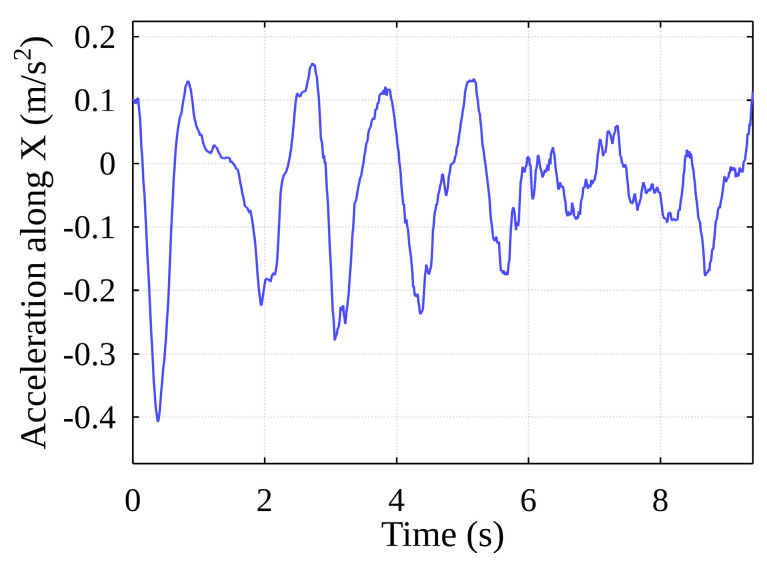
Acceleration of container along X-axis (handling cycle).

The frequency spectrum between 3.5 Hz and 8 Hz captures the oscillations of the container when subjected to external influences, including the so-called safety events, i.e., the impacts of the container on the vertical cell guides. When a container impacts the guides, the direction of its movement changes (see Figure 14).

**Figure 15:**
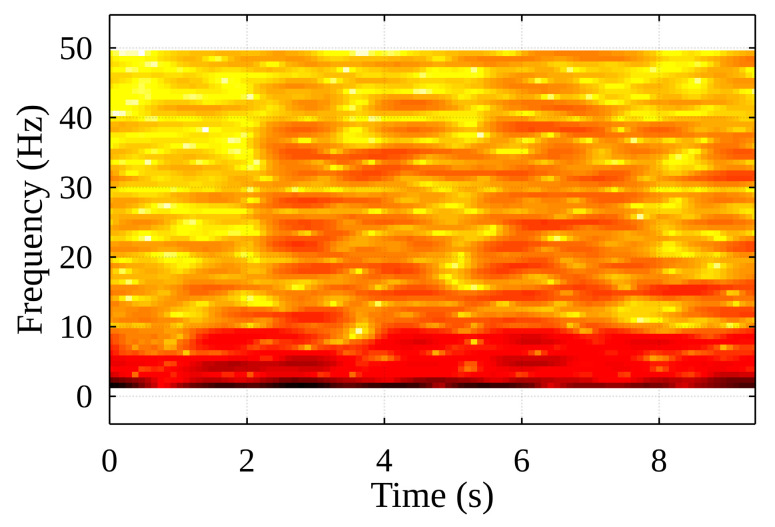
Spectrogram of acceleration along X-axis (handling cycle).

Figure 14 shows sudden changes in the harmonic oscillation of the container, based on the acceleration signal data These changes in the acceleration spectrum “generate” higher frequency components. Comparing this to a typical acceleration spectrogram during the container handling procedure (see Figure 15), it appears highly likely that impacts to the guides occurred at the 3-, 4.5- and 6-s marks (the darker the red shade, the stronger the signal). This assumption is made because the container is unlikely to have experienced any other type of impact in the horizontal plane within the hull of the ship.

The proposed methodology (Figure 11) of detection consists of several steps:

1. signal filtering;
2. detection of peak values in the filtered signal using statistical analysis principles;
3. threshold value selection;
4. exclusion of potential impact moments (events) using a threshold value;
5. isolation of actual events by grouping potential events.

As mentioned, shocks “generate” higher frequency components in the acceleration signal, so using a high-pass filter isolates these oscillations for further analysis. To minimize the distortion of the signal for further analysis, a filter is required here that has a monotonic amplitude response in the passable frequency range, the lowest possible phase distortions, and a constant delay throughout the frequency range. For this purpose, a high-pass FIR filter with the following coefficients was chosen (1):

(1) $$H\left(z\right)=\frac{B\left(z\right)}{A\left(z\right)}=b_{0}+b_{1}z^{-1}+b_{2}z^{-2}+\ldots +b_{N}z^{-N},$$

Here, *N*—filter queue and *b*—coefficients are obtained by using the GNU Octave function “fir1”, as described below.

Arrays of positive and negative ranges are merged into a common range. The following algorithm was used to identify the extremes of half-waves:

(2) $$\left[p,p_{\mathrm{idx}}\right]=\begin{array}{c} M\\ i=1 \end{array}|max\left(\left|a_{f}\left(S_{i}:E_{i}\right)\right|\right),$$

where

*a*_f_—an array of filtered accelerations;
*S*—an array of half-wave start indices;
*E*—an array of half-wave end indices;
M—the number of half-waves;
*p*—values of extremums found;
*p*_idx_—positions of found extremums in an array of filtered accelerations.

**Figure 16:**
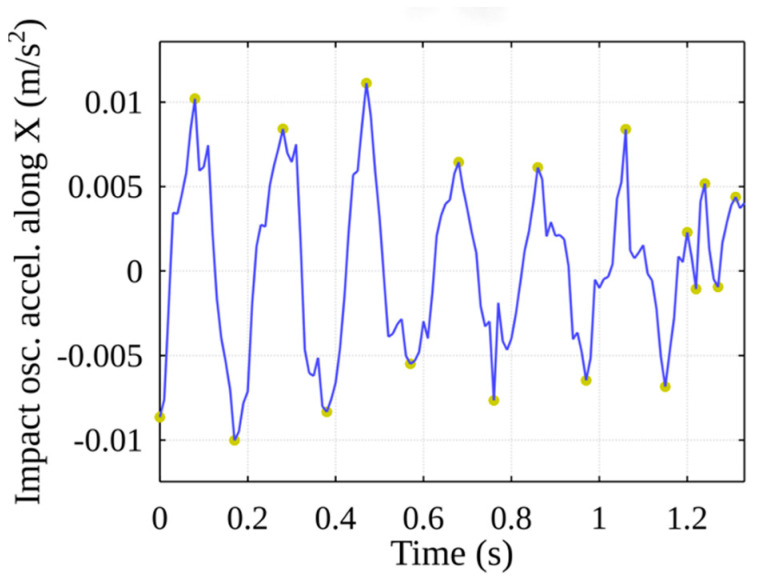
Stage of event detection: peak detection along the X-axis.

A fragment of the results produced by the mathematical algorithm is shown in Figure 16. The blue line represents oscillation impacts and acceleration along the X-axis, and the half-period peaks found by the algorithm are marked in yellow/green circles.

**Figure 17:**
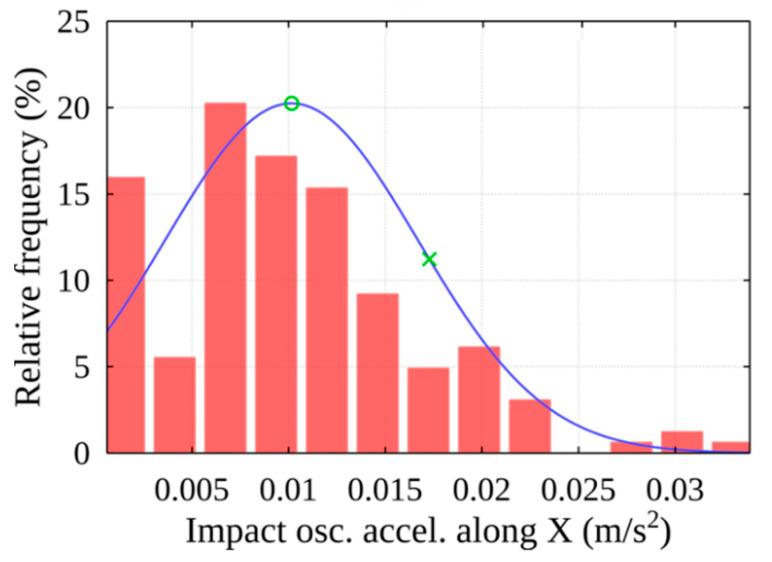
Histogram of peak amplitudes of acceleration along X-axis.

Any peak found in the previous stage may be the result of an impact. The threshold value of the filtered signal is used to select the peaks associated with the impact: peaks with absolute values greater than the threshold indicate potential moments of events. Statistical analysis is used to determine the threshold. It finds the distribution function that best describes the peak values. In its peak area, the most common peak values (see Figure 17), which are marked with a green circle, can be considered noise. The method employs a threshold value for the parameter used, the event frequency, which corresponds to 100% at this point. Progressing in the direction of higher peak amplitudes, as the value of the mentioned parameter decreases, such peaks occur less frequently within the signal. After selecting a certain value of the mentioned parameter (see Figure 17), which is marked with a green cross, we obtain the acceleration threshold value according to the distribution function.

**Figure 18:**
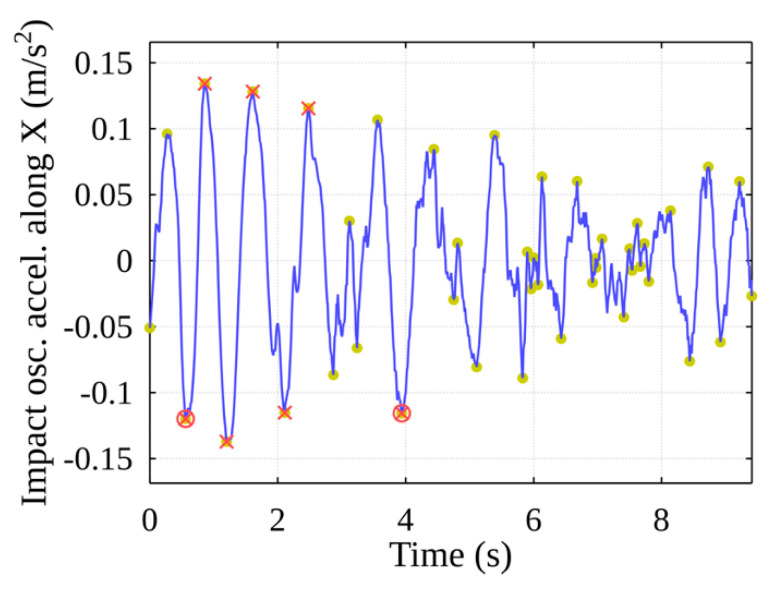
Stage of event detection: event peaks filtered after threshold value is chosen.

Using the obtained acceleration threshold value, the peaks with amplitudes higher than the threshold value are selected from the sample of peaks, thereby identifying the peaks that indicate potential events (see Figure 18), marked with red crosses. Using the developed algorithm, potential events are grouped, resulting in separate shock events, and their beginnings are marked with red circles.

**Figure 19:**
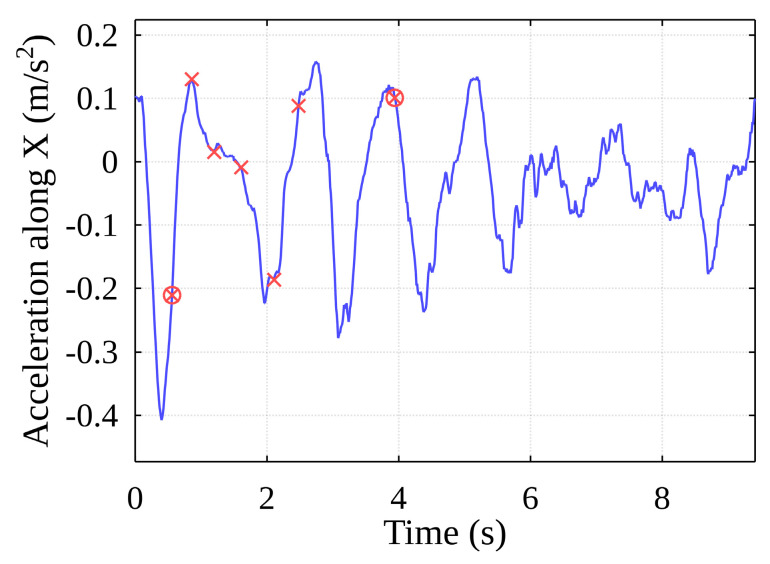
Potential events from unfiltered acceleration data.

Figure 19 shows the original acceleration curve for lifting a container with projections of the events described above from unfiltered acceleration data.

**Figure 20:**
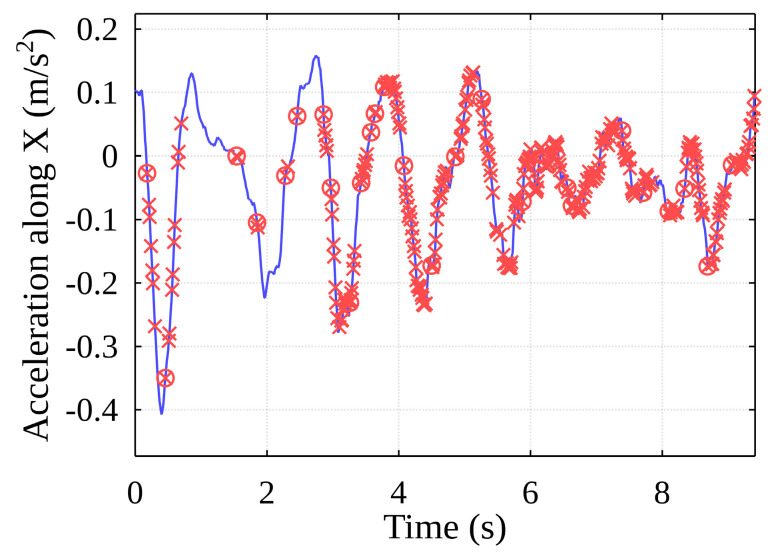
Event peaks are filtered after another threshold value is chosen.

As mentioned, the methodology uses two selectable parameters: the filter corner frequency and the event frequency threshold. Varying these over a wide range generates results that also vary over a wide range (compare, for example, Figure 19 and Figure 20, in which different parameter values were found).

**Figure 21:**
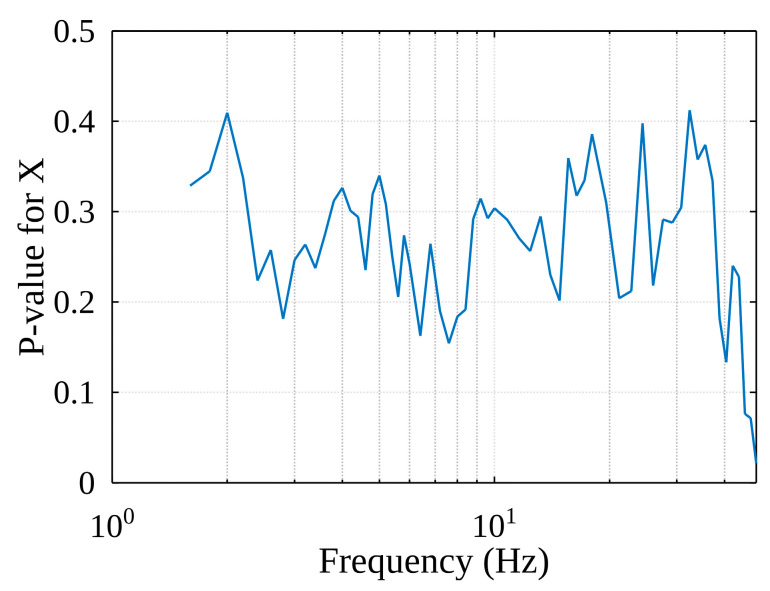
*p*-value of the selected distribution function.

A study was conducted during which the parameter values were changed over a wide range and the results obtained using the method were evaluated. In this study, 17 cases of container lifting were investigated, and the sequence of the obtained values was summarized by calculating their root means square (RMS). As we can see in Figure 21, the *p*-value of the selected distribution function (obtained from the Chi-square goodness-of-fit test) changes as the filter frequency changes: the RMS value does not fall below 0.15 and several local maxima can be seen here. The conclusion is that the filter corner frequency should be chosen according to the local maximum at lower frequencies because, according to the spectral acceleration analysis (see Figure 15), the oscillations of high-amplitude accelerations that are of interest to us occur at lower frequencies.

**Figure 22:**
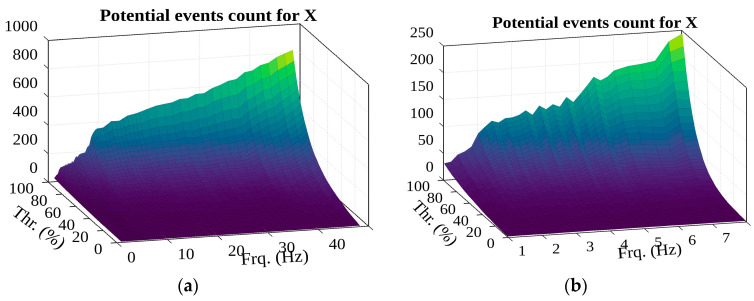
The surface of potential events X f (in the (**a**)—1–49 Hz; (**b**)—1–8 Hz frequency range).

Looking at the number of potential detected events (see Figure 22), it can be observed that increasing the filter frequency decreases the period of acceleration oscillations, and the number of detected peaks for the same period therefore increases. At the beginning of the filter frequency range, at 2 Hz and 8 Hz, there is a jump in the number of events (looking at the extremes); this may be important in further analysis. The shocks that are of interest to us should generate oscillations with high-amplitude accelerations, which, as spectral analysis has shown, are in the low-frequency range. However, the schedule suggests that higher frequencies should be chosen, indicating that this size alone is not a suitable selection parameter.

**Figure 23:**
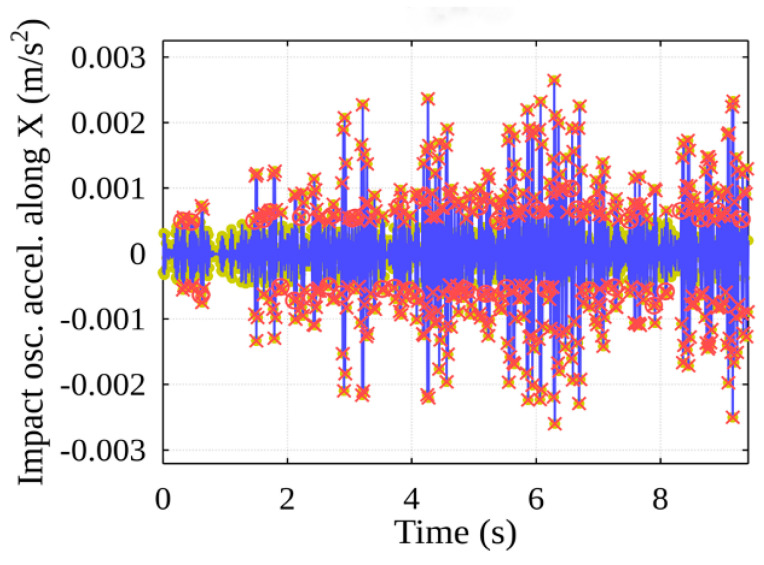
The possible impacts were detected from data of acceleration along the X-axis.

Looking at the number of detected events (see Figure 23), it can be observed that the number of groups of potential events increase as the filter frequency increases. This can be attributed to the fact that, as the frequency increases, the oscillation amplitude of the filtered acceleration decreases and the oscillation bursts increase, meaning the chances of forming more groups is potentially higher.

**Figure 24:**
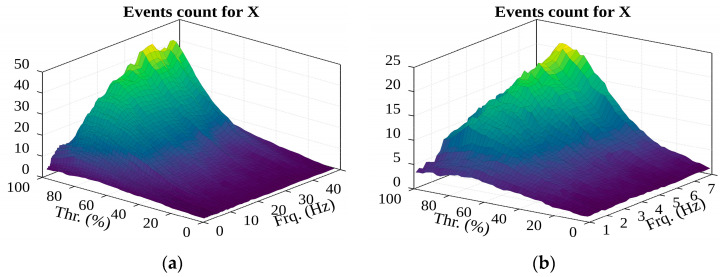
The surface of events (in the (**a**)—1–49 Hz; (**b**)—1–8 Hz frequency range).

In addition, as the filter frequency increases (see Figure 24), the graph shows a sudden jump in the number of recorded events (looking at the extremes) at the beginning of the filter frequency range, and from 2.5 Hz the change stabilizes and becomes linear. This makes it possible to assume that the frequency range of the filter should start from this value in further studies. Assessing the number of detected events leads to the previous erroneous conclusion: the filter frequency and percentage threshold need to be increased. However, it is unlikely that more than 20 serious shocks will occur when the container is lifted, so filter frequency values from 8 Hz should not be used in further studies.

**Figure 25:**
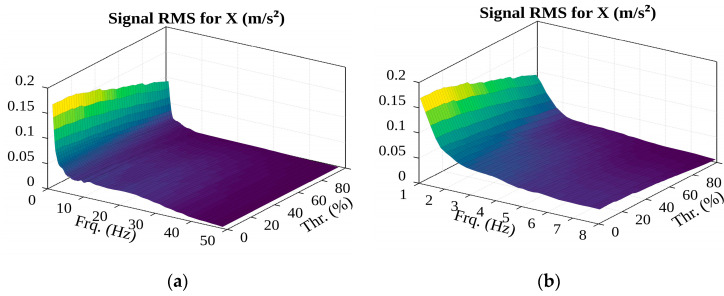
The surface of RMS of potential events amplitudes (in the (**a**)—1–49 Hz; (**b**)—1–8 Hz frequency range).

After calculating the RMS values of the filtered acceleration half-wave associated with each potential event, and then averaging them, a graph is obtained (see Figure 25). The expression used for this purpose is as follows:

$${\bar{a}}_{\mathrm{RMS}}=\frac{1}{N}\left(\sum_{n=1}^{N}\sqrt{\frac{1}{E_{n}-S_{n}+1}\sum_{i=S_{n}}^{E_{n}}a_{i}}\right),$$

where

*a*—acceleration value array;
*N*—the number of half-waves of potential events;
*S*—an array of indices of the half-waves;
*E*—an array of half-wave end indices.

As the filter frequency increases, the mean values decrease. This can be explained by the almost exponential decay of the acceleration components as their frequency increases (see Figure 15). Looking at the results, it appears that the lowest possible filter frequency and percentage threshold should be chosen to obtain good event detection, but this is an unlikely finding.

Thus, a different weighting is needed to select the parameter values. This could be a product of several of the aforementioned quantities.

**Figure 26:**
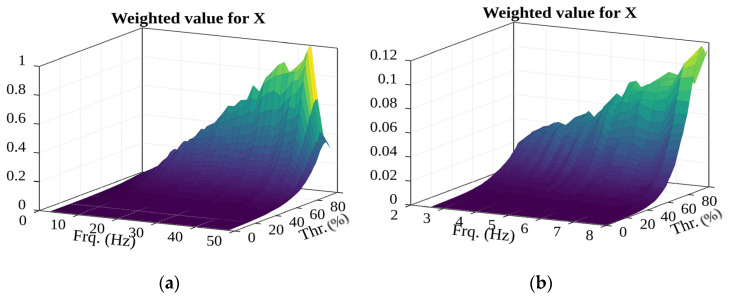
The surface of weighted value of potential events (in the (**a**)—2.6–49 Hz; (**b**)—2.6–8 Hz frequency range).

One option is the product of the number of potential events and the number of events (see Figure 26). However, this case, as before, must be ruled out, as the peak value is in the high-frequency range, and a high percentage threshold would merely lead to noise-induced results being obtained.

**Figure 27:**
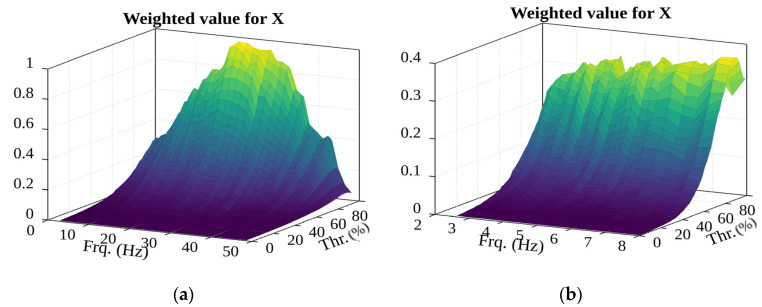
First evaluation of the surface of the weighted value of potential events and RMS (in the (**a**)—2.6–49 Hz; (**b**)—2.6–8 Hz frequency range).

Using the product of the number of potential events and the average RMS of potential events (see Figure 27), we can see that there is no clear value for the selection: the filter frequency and percentage should be as low as possible.

In Figure 27, the results are obtained by multiplying the number of potential events, the number of events, and the average RMS of potential events. This estimate is not appropriate because similar extremes are observed in the high-frequency range and the proposed percentage threshold will “grab” a lot of noise.

**Figure 28:**
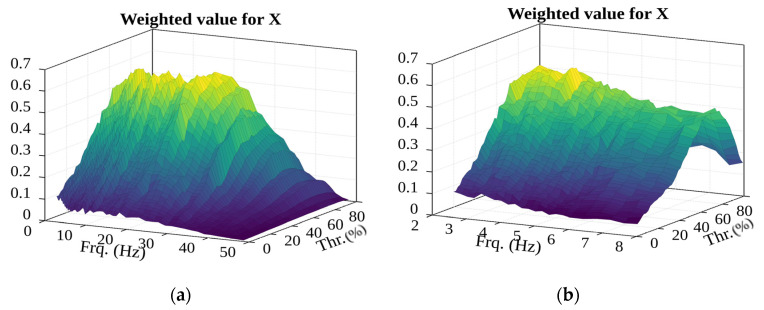
Second evaluation of the surface of the weighted value of potential events and RMS (in the (**a**)—2.6–49 Hz; (**b**)—2.6–8 Hz frequency range).

The product of the number of events and the average RMS of potential events (see Figure 28) seems to fit the estimate. Except for the filter frequency range (described in the paragraph above on the number of detected events), a local extremity is seen, which is in the “reasonably” low filter frequency range and the sufficiently low percentage threshold range: 3.8 Hz and 73%, respectively.

**Figure 29:**
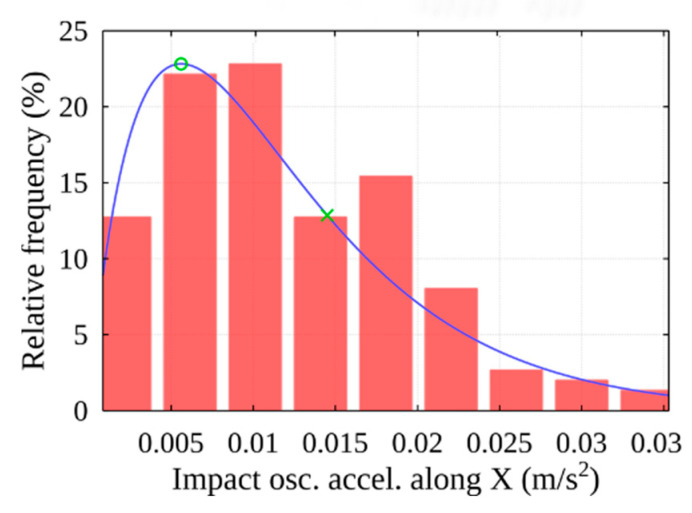
Histogram of peak amplitudes of acceleration along X-axis (using the chosen 73% threshold).

After selecting these values for the methodology, the filtered acceleration signal of one container lift during the sample cycle was obtained, and the selected distribution is given in Figure 29.

**Figure 30:**
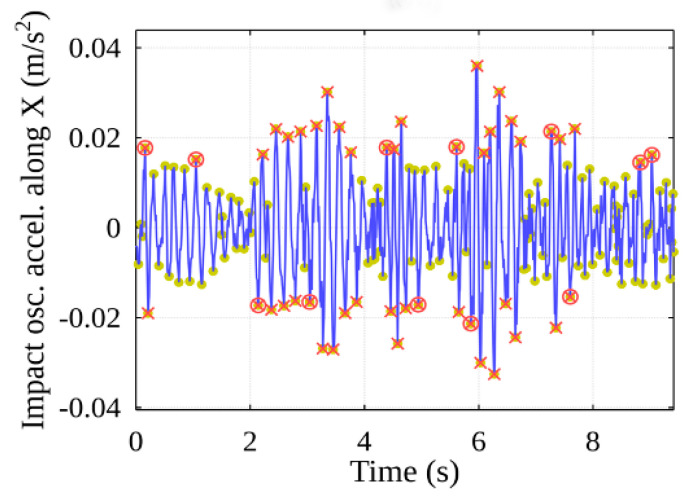
Result of stages of the event detection algorithm.

In this case, the distribution of peak amplitudes is best matched by the gamma distribution function. Using histogram data, the threshold is detected and determined according to the proposed methodology marked as X (see Figure 29), according to the threshold acceleration value of 0.014 m/s^2^ selected according to the distribution function (see Figure 30).

From the total value of potential events (Figure 29, yellow dots), only those for which the peak amplitudes are equal to or greater than the set threshold are selected. In this way, the time of the container contact with the ship’s cell guides is determined, and an event is judged to have taken place (Figure 29, red X).

**Figure 31:**
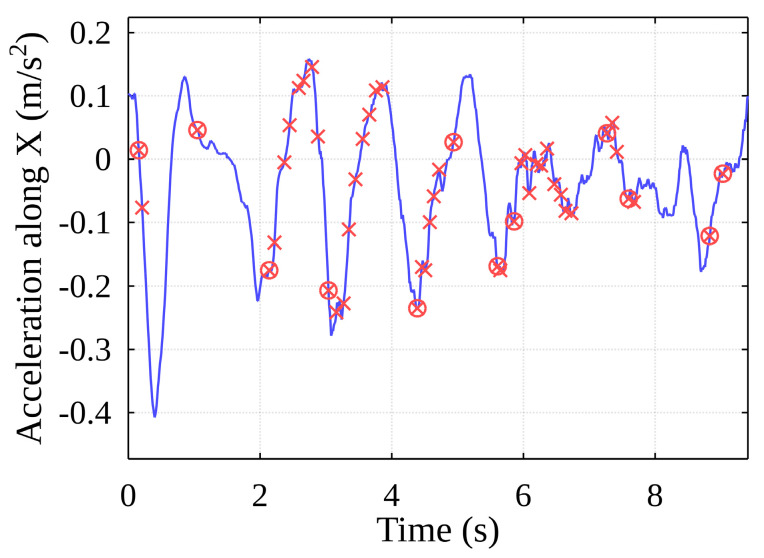
Detected events mapped on data of acceleration along the X-axis.

As the container oscillates horizontally within the free space inside the ship’s hull, it comes into contact with one of the vertical cell guides, after which it can remain in contact with it or collide with another guide. For this reason, the algorithm implements the separation of the above-mentioned main events, the events are grouped, and the algorithm returns the final main event start timestamps (Figure 30, red circles). The event data generated by the algorithm can be mapped to the data recorded by the original sensors (Figure 31).

With this precise experimental measurement, the algorithm detected around 12 main impacts with the vertical cell guides. All of them were detected with a high degree of accuracy using the presented method and technical equipment. The proposed methodology is therefore suitable to be used to detect impacts in an almost real-time manner, if used appropriately and with a sufficient computational device.

## 5. Results and Discussion

The results show that the proposed solution can identify impacts to the vertical cell guides for each hold, and for each individual cell. A combination of the proposed methodology and the developed IoT device can detect critical impacts, filtering the signals according to the proposed threshold detection rule and logging the data for future use by the managers of the operations or sending critical alert information to the operators on site for predictive maintenance and possible damage evaluation, utilizing best practices from the adoption of the most suitable industrial wireless communication technologies. The convenience and effectiveness of the proposed vertical cell guide damage detection system could provide great advantages in largescale environments, enabling statistics to be gathered for each ship, while also proving logistics companies with critical information about possible damages to the goods inside the containers during the same impacts, drastically increasing the viability of these operations.

Each impact event takes place over tens of milliseconds, while sliding events that occur along the vertical cell guides occur at time scales of a few seconds. Furthermore, the measured signals are contaminated by noise components from several other naturally occurring and unnatural processes extraneous to the natural motion of the container, including the quay crane and spreader dynamics, as well as environmental and electronic noise. This vast disparity in time scales, as well as the issues with signal contamination, pose serious signal processing and de-noising challenges for conventional methods, operating in harsh working conditions. However, theoretically, these challenges can be effectively addressed through a combination of several separate system, with novel and known algorithms in a unified methodology.

We tested several frequency ranges for the weighted values of potential events and RMS, and selected the best, as determined via the selective analysis provided in the methodology section. When the containers are lifted out of the ship’s holds, they oscillate at a harmonic frequency. The frequency range between 0.4 Hz and 2.5 Hz has been determined for the specific cases of our physical experiment in the port of Klaipeda. However, the free space for the containers to move is very limited by the surrounding infrastructure and the vertical cell guides, meaning the contacts of the container with the guides generates oscillations with higher frequency components. In our case study, our proposed impact detection methodology analyzed the oscillation frequency range starting from 3.8 Hz. After setting the threshold at 78%, our method was able to detect the known impacts, which occurred an average of 12.8 times, without filtering the less important sliding effects that sometimes occur with each new impact. After close examination of other additional case studies, also shown in Figure 8, we obtained almost identical results; with the threshold set at 78%, the number of detected impacts varied from 6.8 to 14.6, due to different depths of the holds.

Our results indicate that it is possible to detect not only the impacts themselves but also the exact place and moment at which they occurred. This methodology was developed using historical data from a case study for true threshold detection. The methodological efficiency could be refined if a larger data set were used during the evaluation process, consisting of full-cycle operations, along with modified standards for the entire ship. In this way, if applied globally, it would be possible to detect a vast range of security events, monitoring the containers and the cargo, the handling infrastructure, and the different transport means. This solution can be used primarily in predictive maintenance and other decision support systems. However, for such an application, this methodology would need to be refined from the perspective of a single handling cycle for events detection. This proposed solution can be used for the risk assessment of a single container hold. The main advantage of this type of system its ability to track repeated impacts that occur with each individual cell. This allows sufficient data to be collected to enable adequate decision-making using the proposed methodology.

## 6. Conclusions

The results show that the proposed methodology can identify impacts to the vertical cell guides. In our case study, we managed to detect 12.8 impacts on average during the handling of the container from the bottom of the hold. The proposed methodology adopted a 78% threshold, but the origin of the rest of the impacts still remains to be analyzed. The acquired data from the sensory unit and the impact results were presented to the personnel of the terminal, and the common discussions highlighted the strategic problem of vertical cell guide wear resulting from impacts, an issue that had previously received inadequate attention from the company and its personnel. Our research aim with the container terminal company was to detect these impacts as they continually occur within the same holds throughout the full unloading cycle of a ship, classifying different events and types of impacts (pattern detection), and providing the personnel of the terminal with more in-depth and reliable information about these critical incidents.

Our future research will further examine the following aspects: increased reflection on the security and synchronization of the physical space during the entire process, taking into consideration other berths and transport means; and increased focus on any correlations between historical and real-time sensory data in order to improve the quality of the estimation results and the level of fidelity for each individual handling process. Regarding the IoT device, we intend to further optimize the data sampling technique locally, improving the Edge computing paradigm, while lowering its power consumption and computational strain, and promoting efficient wireless communication innovations. A new real-time monitoring system will be developed based on the existing prototype and methodology, and may be integrated into the Klaipeda city LKAB “Smelte” container terminal management system (as a sub-section of predictive maintenance and repairs).

Finally, it is necessary to qualify the degree of detection accuracy with regards to the severity of any impacts with the vertical cell guides in order to support intelligent decision-making for the entire port operation.

## Data Availability

Not applicable.
